# Safety and Performance of a Cell-Impermeable Endoprosthesis for Hemodialysis Vascular Access Outflow Stenosis: A Brazilian Multicenter Retrospective Study

**DOI:** 10.1007/s00270-024-03790-1

**Published:** 2024-07-02

**Authors:** Leonardo de Oliveira Harduin, Thiago Almeida Barroso, Julia Bandeira Guerra, Márcio Gomes Filippo, Leonardo Cortizo de Almeida, Brunno Ribeiro Vieira, Renata Silveira Mello, Adriano Martins Galhardo, Jorge Paulo Strogoff-de-Matos

**Affiliations:** 1 Liv Care Centro Clínico, Niterói, Rio de Janeiro 24220-002 Brazil; 2 Afya Hospital Dia, Brasília, Distrito Federal Brazil; 3 Image Department, Hospital Niterói Dor and Centro Clínico LIVCARE, Niterói, Rio de Janeiro Brazil; 4 Vascular Surgery Service, Hospital Universitário Clementino Fraga Filho (HUCFF), Universidade Federal do Estado do Rio de Janeiro (UFRJ), Rio de Janeiro, Rio de Janeiro Brazil; 5 Vascular Surgery Service, Hospital Ana Nery, Salvador, Bahia Brazil; 6 Instituto Nacional de Traumatologia e Ortopedia, Rio de Janeiro, Rio de Janeiro Brazil; 7 Vascular Surgery Service, Hospital Niterói Dor, Niterói, Rio de Janeiro Brazil; 8 Divisão de Nefrologia, Departamento de Medicina, Faculdade de Medicina, Universidade Federal Fluminense, Niterói, Rio de Janeiro Brazil

**Keywords:** Hemodialysis, Cell-impermeable endoprosthesis, Covered stent, Stent graft, Vascular access, Stenosis

## Abstract

**Purpose:**

To evaluate the safety and performance of Wrapsody™, a cell-impermeable endoprosthesis (CIE), for treating hemodialysis vascular access outflow stenosis.

**Materials and Methods:**

Investigators retrospectively analyzed 113 hemodialysis patients treated with a CIE (11/2021–12/2022) across four centers in Brazil. De novo or restenotic lesions were treated. The primary efficacy outcome measure was target lesion primary patency (TLPP) at 1, 3, 6, and 12 months; the primary safety outcome measure was the absence of serious local or systemic adverse events within the first 30 days post-procedure. Secondary outcome measures included technical and procedural success, access circuit primary patency (ACPP), and secondary patency at 1, 3, 6, and 12 months post-procedure.

**Results:**

Thirty-nine patients (34.5%) had thrombosed access at the initial presentation, and 38 patients (33.6%) presented with recurrent stenosis. TLPP rates at 1, 3, 6, and 12 months were 100%, 96.4%, 86.4%, and 69.7%, respectively. ACPP rates were 100% at 1 month, 89.2% at 3 months, 70.9% at 6 months, and 56.0% at 12 months. The target lesion secondary patency rates at 1, 3, 6, and 12 months were 100%, 97.3%, 93.6%, and 91.7%, respectively. In the adjusted multivariate Cox regression analysis, male sex and endoprosthesis with diameters of 10, 12, 14, and 16 mm were associated with improved primary patency rates. No localized or systemic serious adverse event was observed through the first 30 days post-procedure.

**Conclusion:**

The CIE evaluated in this study is safe and effective for treating peripheral and central outflow stenoses in hemodialysis vascular access.

**Level of Evidence:**

Level 2b, cohort study.

**Graphical Abstract:**

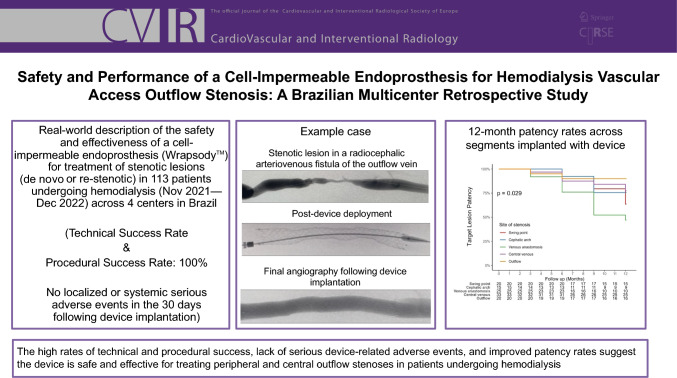

**Supplementary Information:**

The online version contains supplementary material available at 10.1007/s00270-024-03790-1.

## Introduction

Arteriovenous fistulas (AVF) and arteriovenous grafts (AVG) are critical for long-term vascular access for hemodialysis patients. However, complications (e.g., stenosis/occlusion) leading to impaired vascular access are inevitable [1]. Intimal hyperplasia and fibrosis are common causes of stenosis/occlusion in AVFs and AVGs [2–4], unlike arterial and venous territories where atherosclerosis disease and compressive syndromes, respectively, are the main causes of stenosis/occlusion [5, 6].

Recently, Wrapsody™ (Merit Medical Systems, Inc., South Jordan, Utah, USA), a novel cell-impermeable endoprosthesis was developed to manage stenosis/occlusion in the dialysis outflow circuit [7]. Compared to conventional stent grafts, this device has been constructed with reduced end-row radial force to minimize edge stenosis and a cell-impermeable layer within the polytetrafluorethylene (PTFE) to limit trans-graft cellular migration [8]. Moreover, the device is available in larger diameters compared to commercially available stent grafts, which is particularly beneficial for hemodialysis access applications, where accommodating larger-diameter vessels is crucial for ensuring long-term patency.

Since the publication of the first-in-human results [7], limited information is available regarding the device’s efficacy and safety. This study was conducted to address this knowledge gap by describing the device’s safety and effectiveness in a real-world patient population.

## Materials and Methods

### Patient Selection and Study Design

In this retrospective study, hemodialysis patients (≥ 18 years) with vascular access outflow stenosis (11/2021–12/2022) who were treated using the Wrapsody Cell-Impermeable Endoprosthesis (Merit Medical Systems, Inc., South Jordan, Utah, USA) were eligible for inclusion. Patients were required to have had angioplasty for the salvage of AVFs or AVGs and significant angiographic stenosis (luminal narrowing ≥ 50%) that was diagnosed via doppler vascular ultrasound and confirmed via angiography during the intervention procedure. All patients included in this study presented with stenosis accompanied by clinical signs of dysfunctional dialysis access (e.g., variation in thrill/bruit, difficult cannulation, recirculation, edema, excessive bleeding from the venipuncture site, or thrombosis [9]). Additional inclusion criteria were target lesions < 10 cm in length, and vessels with diameters ranging from 4.6 to 14.4 mm. Patients with lesions extending across the elbow were excluded.

### Study Device

The device is a flexible, self-expanding, tri-layer cell-impermeable endoprosthesis. The innermost PTFE layer was designed to limit inflammation and thrombus formation, the cell-impermeable middle graft layer prevents transmural cellular migration [8], and a standard biocompatible expanded PTFE outer layer allows for necessary tissue ingrowth to prevent stent migration.

The device is available in diameters ranging from 6 to 16 mm and in lengths ranging from 30 to 125 mm, enabling the treatment of vessels ranging from 4.6 to 14.4 mm in diameter. The delivery catheter is available in lengths of 80 and 120 cm and is compatible with sheath sizes ranging from 8 to 14-French. The device is indicated for the treatment of stenosis or occlusion within central veins, as well as the dialysis outflow circuit of an AVF or AVG.

### Study Treatment and Follow-Up

Two vascular surgeons and two interventional radiologists performed the procedures while patients were under local anesthesia and sedation. Access was via the femoral vein when addressing graft-vein anastomosis without thrombosis, or when there was no vein segment measuring ≥ 9 mm in diameter within the AVF. This approach minimizes the risk of stenosis within the site of the access after sheath removal and closure of the site puncture with a hemostatic stitch. In cases with thrombosis at initial presentation, thrombectomy, with complete flow restoration, was performed prior to angioplasty. The choice of the access site for thrombectomy was based on the standard operating procedures at each site. Open thrombectomy was performed with a Fogarty catheter; endovascular thrombectomy was performed via femoral access or directly within the AVF or AVG.

If thrombosis was detected at initial presentation, the same vein segment used during the thrombectomy procedure was used for subsequent outflow stenosis angioplasty.

A 0.035-inch hydrophilic guidewire was used to cross the lesion. The area was then pre-dilated using a high-pressure balloon chosen at the operator’s discretion (Conquest® [BD, Franklin Lakes, New Jersey, USA]; Mustang® [Boston Scientific, Marlborough, Massachusetts, USA]; Fortrex™ [Medtronic, Minneapolis, Minnesota, USA]; Atlas™ [BD, Franklin Lakes, New Jersey, USA]; Cronus® HP [Nipro Medical, Bridgewater, New Jersey, USA]). The balloon was inflated to a pressure level that ensured complete expansion of the pre-dilation balloon. After pre-dilation and angiographic analysis, the device was deployed.

To ensure proper anchoring and contact with the vessel wall, the cell-impermeable endoprosthesis was placed in a 10–25% oversized configuration within the distal segment of the stenosis (compared to the adjacent healthy segment). This configuration included ≥ 1 cm overlap with the healthy vessel or synthetic graft. Devices were then post-dilated using a balloon, with the balloon size not exceeding the diameter of the device. After the procedure, patients were administered antiplatelet and/or anticoagulant therapy. Two centers administered both aspirin and clopidogrel (60 days at one center; 30 days at another), whereas the other two centers exclusively prescribed a 30-day course of either aspirin or clopidogrel.

Physical examinations were scheduled at 1, 3, 6, and 12 months post-procedure, with additional visits arranged as needed to address any issues related to access circuit dysfunction. Follow-up times enabled comprehensive assessments during the interval when conventional interventions tend to fail.

Physical examinations and dialysis parameters evaluated included the presence of thrill, prolonged bleeding, elevated venous pressure, pulsatility, difficulties in needle cannulation, and inadequacy of the hemodialysis session—identified by low Kt/V values, where values are determined by urea volume clearance (K) during the dialysis session (t) divided by the distribution volume of urea (V), as well as observations for edema in the hand, arm, neck, and trunk. Assessments were conducted to detect any discomfort associated with the dialysis circuit, respiratory and neurological symptoms, skin condition changes, and adverse effects. If a patient presented with symptoms of dysfunction during the physical examination, and a new stenosis or restenosis was suspected, a Duplex ultrasound or angiogram was performed to confirm the diagnosis.

### Study Outcome Measures

Study measures included patient characteristics, access failure causes, access patency, need for reintervention, proper access functioning, factors predicting procedure success, complication rates, and mortality rates.

Clinical outcomes were retrospectively evaluated 12 months following device placement. The primary and secondary study outcome measures are listed in Table 1; definitions of study measures align with the Society of Interventional Radiology reporting standards [10].

**Table 1:** Study outcome measures

Outcome measure type	Outcome measure description	Outcome measure definition
Primary outcome measure	Target lesion primary patency at 1, 3, 6, and 12 months	The time between device placement and any subsequent intervention within the region where the device was implanted or access circuit thrombosis
Primary safety outcome measure	30-day safety	Freedom from localized or systemic serious adverse events through 30 days post-procedure
Secondary outcome measure	Technical success	Successful deployment of the device at the intended location
Secondary outcome measure	Procedural success	Achieving < 30% residual stenosis following the completion of the procedure accompanied by the resolution of the preprocedural clinical indicators of access dysfunction
Secondary outcome measure	Access circuit primary patency at 1, 3, 6, and 12 months	The time spanning from device placement to the initial intervention anywhere within the access circuit or in the event of access circuit thrombosis
Secondary outcome measure	Target lesion secondary patency at 1, 3, 6, and 12 months	The time from initial device placement until the AVG or AVF was abandoned

AVF, arteriovenous fistula; AVG, arteriovenous graft

### Statistical Analysis

Continuous variables were summarized using mean ± standard deviation, categorical variables were reported as frequencies, counts, and percentages. Patency analysis was performed with the Kaplan–Meier method and, when applicable, compared using the log-rank test. Hazard ratios for patency loss were estimated by Cox proportional hazards regression with 95% confidence intervals (CI). Initially, univariate analysis was performed for each demographic, clinical, and anatomical variable. Subsequently, only those variables that showed *P*-values < 0.20 in the univariate assessment were included in the multivariate model. *P*-values < 0.05 were considered statistically significant. Statistical analyses were conducted using R version 4.1.0 (R Core Team, 2021).

## Results

### Patient and Lesion Characteristics

A total of 113 patients were analyzed (Table 2). Most patients were male (53.1%) and aged < 65 years (54.0%). The most prevalent comorbidity was hypertension (82.3%). Thirty-nine patients (34.5%) had thrombosed access at the initial presentation. Thirty-eight patients (33.6%) presented with recurrent stenosis, and 75 patients (66.4%) presented with de novo lesions.

**Table 2:** Patient demographics and concurrent medical conditions

Variable		Patient (*N* = 113)	%
Age	< 65 years	61	54.0
	≥ 65 years	52	46.0
Sex	Female	53	46.9
	Male	60	53.1
Stenosis type	Cephalic arch	20	17.7
	Swing point	15	13.3
	Venous anastomosis	25	22.1
	Central venous stenosis	33	29.2
	Outflow	20	17.7
Restenosis	De novo lesion	75	66.4
	Recurrent/restenotic lesion	38	33.6
Thrombosis	No	74	65.5
	Yes	39	34.5
Hypertension	No	20	17.7
	Yes	93	82.3
Diabetes mellitus	No	66	58.4
	Yes	47	41.6
Coronary heart disease	No	85	75.2
	Yes	28	24.8
Smoker	No	95	84.1
	Yes	18	15.9
Stroke	No	107	94.7
	Yes	6	5.3
Other	No	101	89.4
	Yes	12	10.6

Other comorbidities include systemic lupus erythematosus, n = 3; polycystic kidney disease, n = 4; glomerulonephritis, n = 2; nephrolithiasis, n = 3

The types of access treated were AVGs in 33.6% of patients, brachiocephalic AVFs in 36.3%, and brachiobasilic AVFs in 22.1% of patients (Table 3). Most patients received devices between 8 and 9 mm in diameter and up to 75 mm in length. The procedures were performed through the femoral vein in 62 patients (54.9%).

**Table 3:** Dialysis access circuits and stent graft characteristics

Variable		Patient (*N* = 113)	%
Arteriovenous access	Brachiocephalic	41	36.3
	Basilic transposition	25	22.1
	Arteriovenous graft	38	33.6
	Radiocephalic	5	4.4
	Ulnar basilic	2	1.8
	Radial basilic	2	1.8
Stent diameter	6 and 7 mm	26	23.0
	8 and 9 mm	39	34.5
	10 and 12 mm	19	16.8
	14 and 16 mm	29	25.7
Stent length	Up to 75 mm	64	56.6
	100 and 125 mm	49	43.4
Angioplasty post stent deployment	No post ballooning or ballon with a smaller diameter than the implanted stent	19	16.8
	Balloon with the same diameter as the implanted stent	94	83.2

### Procedural and safety outcomes

**Fig. 1:**
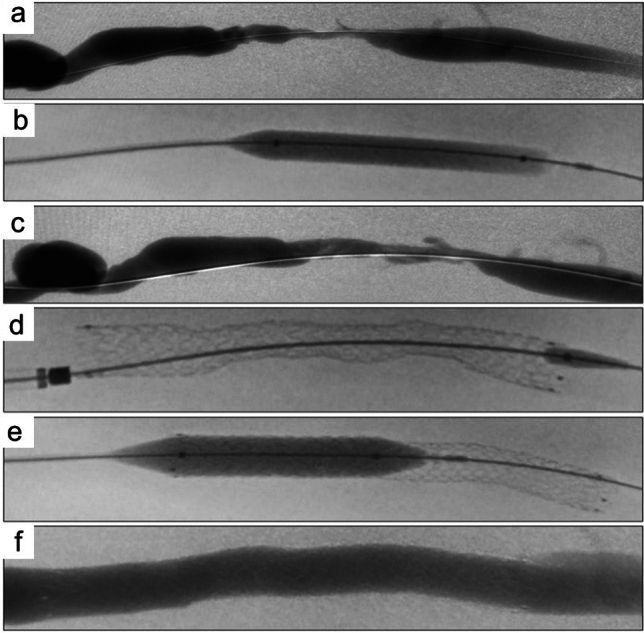
Example of procedural outcomes in a patient with a proximal radiocephalic arteriovenous fistula with stenosis at the outflow vein. **a** Angiography of a basilic outflow vein with severe stenosis; **b** pre-dilation with a 7 × 60 mm high-pressure balloon; **c** intraoperative angiography with important residual stenosis; **d** after 10 × 100 mm deployment; **e** post-dilation with a 10 × 40 mm high-pressure balloon; **f** final angiography after implantation of the 10 × 100 mm endoprosthesis

Technical and procedural success rates were 100%. Figure 1 displays the procedural outcomes of an example case. No serious adverse events were observed locally or systemically during the first 30 days post-procedure. The 10 deaths that occurred during the study period were all attributed to cardiovascular causes; none were related to the device. Access was abandoned in five patients because of infection, one patient was referred for kidney transplantation.

### Performance Outcomes

**Fig. 2:**
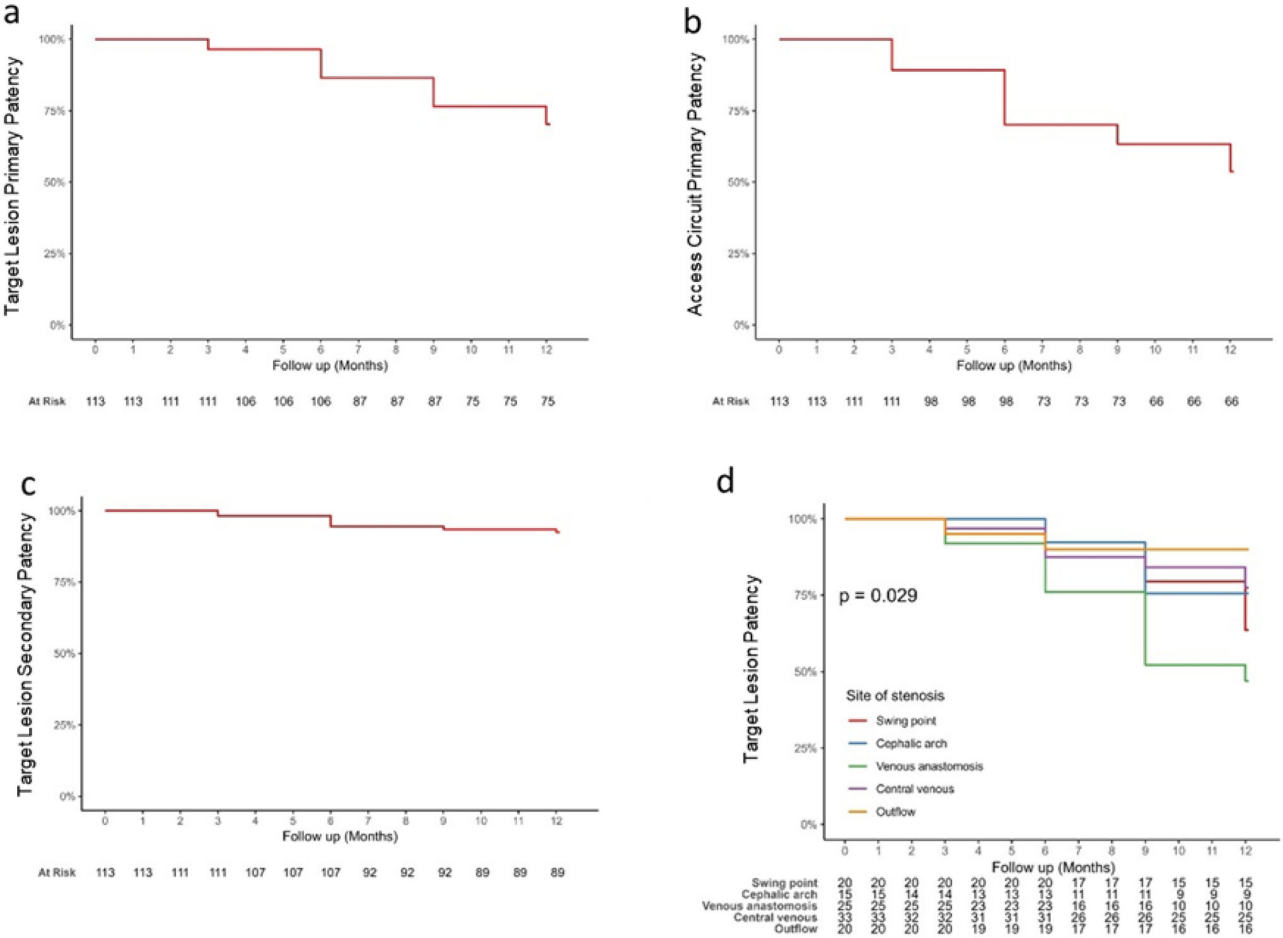
Patency rates. **a** The target lesion primary patency rates were 100% at 1 month, 96.4% at 3 months, 86.4% at 6 months, and 69.7% at 12 months; **b** the access circuit primary patency rates were 100% at 1 month, 89.2% at 3 months, 70.9% at 6 months, and 56.0% at 12 months; **c** the target lesion secondary patency rates at 1, 3, 6, and 12 months were 100%, 97.3%, 93.6%, and 91.7%, respectively; **d** target lesion patency by segments 12 months after device implantation

TLPP rates at 1, 3, 6, and 12 months were 100%, 96.4%, 86.4%, and 69.7%, respectively (Fig. 2a). ACPP rates were 100% at 1 month, 89.2% at 3 months, 70.9% at 6 months, and 56.0% at 12 months (Fig. 2b). The target lesion secondary patency rates at 1, 3, 6, and 12 months were 100%, 97.3%, 93.6%, and 91.7%, respectively (Fig. 2c). A significant difference was observed in the survival distributions across segments in the target lesion patency rates 12 months after device implantation (central vein: 76.7%; basilic swing point: 75.0%; venous graft anastomosis: 45.0%; outflow segments: 88.9%; cephalic arch: 63.2%; *p* = 0.029; Fig. 2d).

During the follow-up period, 30 patients required target lesion reintervention to maintain patency. In all cases, restenosis occurred at the endoprosthesis edge (Fig. S1). Eight patients had their access abandoned due to the failure to recanalize the non-target lesion; of these cases 7 occurred due to the inability to cross the non-target lesion and perform a thrombectomy (4 lesions proximal to the device, 2 lesions in the cannulation zone, and 1 lesion in the juxta-anastomosis segment), the remaining case was abandoned due to repeated thrombosis without stenosis in the circuit.

In the multivariate Cox regression analysis (Table 4), with adjustment for sex, age, diabetes status, fistula type, length and diameter of the device, balloon diameter, stenosis site, thrombosis at initial presentation, and the presence of recurrent lesions, male sex was associated with improved primary patency rates (hazard ratio: 0.36 [95%CI: 0.14, 0.89]; *p* = 0.028). The 6-month primary patency rate in female patients was 78.4% vs. 93.2% in males, and the 12-month primary patency rate in females was 62.5% vs. 76.5% in males (Table S1). Devices with diameters of 10, 12, 14, and 16 mm were associated with improved primary patency rates.

**Table 4:** Cox regression analysis for prediction of 12-month vascular access primary patency failure

Variable	Unadjusted model			Adjusted model		
	HR	95% CI	*P*-value	HR	95% CI	*P*-value
Male sex	0.56	(0.27–1.10)	0.106	0.36	(0.14–0.89)	0.028
Age (≥ 65 years)	1.10	(0.55–2.30)	0.753	1.61	(0.63–4.08)	0.320
Diabetes	0.63	(0.30–1.30)	0.227	0.44	(0.18–1.08)	0.073
Thrombosis	1.90	(0.95–3.90)	0.069	0.62	(0.25–1.52)	0.296
Recurrent lesion	1.10	(0.55–2.40)	0.711	1.45	(0.52–4.05)	0.481
Other stenosis	1.50	(0.72–3.00)	0.297	1.89	(0.85–4.19)	0.119
Arteriovenous graft	2.70	(1.30–5.50)	0.006	3.55	(0.89–14.16)	0.072
Same diameter balloon	2.10	(0.65–7.10)	0.209	1.90	(0.53–6.82)	0.326
Length 100 and 125 mm	1.90	(0.94–3.90)	0.073	2.78	(0.97–7.95)	0.056
Diameter 8 and 9 mm	0.83	(0.38–1.84)	0.651	0.83	(0.29–2.34)	0.719
Diameter 10 and 12 mm	0.10	(0.01–0.78)	0.028	0.08	(0.01–0.91)	0.042
Diameter 14 and 16 mm	0.35	(0.12–1.01)	0.053	0.10	(0.01–0.89)	0.039
Swing point	0.66	(0.17–2.50)	0.544	1.71	(0.36–8.03)	0.498
Venous anastomosis	1.81	(0.71–4.60)	0.214	1.01	(0.18–5.49)	0.995
Central vein	0.62	(0.22–1.80)	0.372	3.56	(0.60–21.12)	0.162
Outflow	0.27	(0.06–1.30)	0.106	0.20	(0.03–1.23)	0.082

CI, confidence interval; HR, hazard ratio

## Discussion

Findings from this retrospective analysis demonstrated that the cell-impermeable endoprosthesis effectively maintained vascular access in hemodialysis patients and was associated with a favorable safety profile. Over half (55%) of the procedures were performed via transfemoral approach. Considering that the required sheath size ranged from 8 to 14-French, this approach avoided direct fistula or graft access, which helped to reduce the risk of stenosis and thrombosis at the puncture site when larger sheaths were required. Moreover, this approach minimized radiation exposure to the physician.

The 6- and 12-month TLPP rates were 86.4% and 69.7%, respectively. Although these rates are lower than the TLPP rates reported in the first-in-human study of the device (6-month TLPP: 97.7%, 12-month TLPP: 84.6%) [7], differences in patient selection criteria may explain this discrepancy. The first-in-human study [7] excluded patients with thrombosed hemodialysis access and secondary lesions, which may have biased results toward a more favorable outcome as thrombosis is often associated with poorer primary patency rates following angioplasty [11, 12]. By including patients with thrombosed hemodialysis access and secondary lesions, the present study offers a more realistic representation of the device’s performance across a spectrum of cases, including challenges typically encountered in clinical practice.

The patency rates in this study were higher than rates following treatment with percutaneous transluminal angioplasty [13, 14], or other types of stent grafts [13, 15–17] with the same indication. For example, in the current study, there was a minimum 26% improvement in the 6-month TLPP within the cephalic arch compared to rates reported with other stent grafts [18, 19]. At 12 months, the patency rate of the endoprosthesis used in this study within the cephalic arch (63.2%) was comparable to a previously reported rate [18]. In the outflow segment, the endoprosthesis assessed in this study achieved a 6-month TLPP rate of 90%, a performance that exceeds the outcomes observed with other covered stents in the same segment [20, 21]. Notably, the patency rate remained high (88.9%) at 12 months.

The 6-month TLPP rate of 76% in the venous graft anastomosis segment aligns with prior evidence [22] and surpasses the patency rates reported for other stent grafts [13, 16]. Although this rate decreased to 45% at 12 months, it is comparable to rates reported in the same segment treated with other stent grafts [17, 22]. Moreover, the 12-month performance of the device in this segment is higher than what has been observed with percutaneous transluminal angioplasty [23]. This suggests that the endoprosthesis used in this study offers a more durable solution for treating stenosis at the graft-vein anastomotic site.

TLPP rates of 92.3% at 6 months and 75.0% at 12 months for the basilic swing point in the present study aligns with prior evidence showing primary patency rates of 57% and 40% at 6 and 12 months, respectively [15]. In central veins, TLPP rates of 87.5% at 6 months and 76.7% at 12 months were observed. These rates are higher than those reported in other studies that included treatment of central venous stenosis [24–30]. A primary limitation when addressing central venous stenosis with covered stents is their size availability [31]. While other covered stents have been used to treat dysfunctional vascular access [31], the device used in this study is currently the only one available in sizes up to 16 mm and the only one evaluated in patients with central venous stenosis [7]. The high patency rates observed in central veins in our cohort, along with enhanced primary patency rates in patients treated with devices with diameters of 10, 12, 14, and 16 mm, suggest that the device can effectively manage difficult cases.

In this study, age, diabetes status, fistula type, length of the device, balloon diameter, stenosis site, thrombosis at initial presentation, and the presence of recurrent lesions did not affect the patency associated with the device. Conversely, being female was associated with a higher risk of patency loss. This may be due to anatomical differences, as women are typically smaller than men and often have veins with smaller diameters, as well as differences in vascular physiology and hormonal variations between males and females [32, 33]; however, additional investigation is warranted.

This study was subject to certain limitations. There was no standardized protocol for the intervention and, therefore, no predefined indications for device implantation. Moreover, as this study did not include a control group, direct comparisons regarding the magnitude of the clinical benefits associated with the device could not be made. Nevertheless, this analysis extends the limited body of evidence for this device and is, to our knowledge, the first study to provide results on the device’s safety and performance in clinical practice.

## Conclusion

The 12-month outcomes from this study suggest that this cell-impermeable endoprosthesis is a viable treatment for stenotic lesions within the hemodialysis access outflow circuit.

### Supplementary Information

Below is the link to the electronic supplementary material.

Supplementary file1 (DOCX 1071 kb)
