# Supplementary material for: Safety and Performance of a Cell-Impermeable Endoprosthesis for Hemodialysis Vascular Access Outflow Stenosis: A Brazilian Multicenter Retrospective Study
Source: Cardiovasc Intervent Radiol. 2024 Jul 2;47(8):1057–65. doi: 10.1007/s00270-024-03790-1 (PMC11303476; doi:10.1007/s00270-024-03790-1)
Supplement: Supplementary file 1 — Supplementary file1 (DOCX 1071 kb) [file 270_2024_3790_MOESM1_ESM.docx]

# Supplemental Material

## Figure S1. Angiographic images of restenosis at the edges of the device.

a) Intravascular ultrasound without intimal hyperplasia in the middle of the device; b) Intimal hyperplasia in the proximal edge; c) intimal hyperplasia in the proximal edge; d) A brachiocephalic arteriovenous fistula with the device in the cephalic arch 11 months post-procedure. The angiography has a stenosis at the proximal edge of the device.


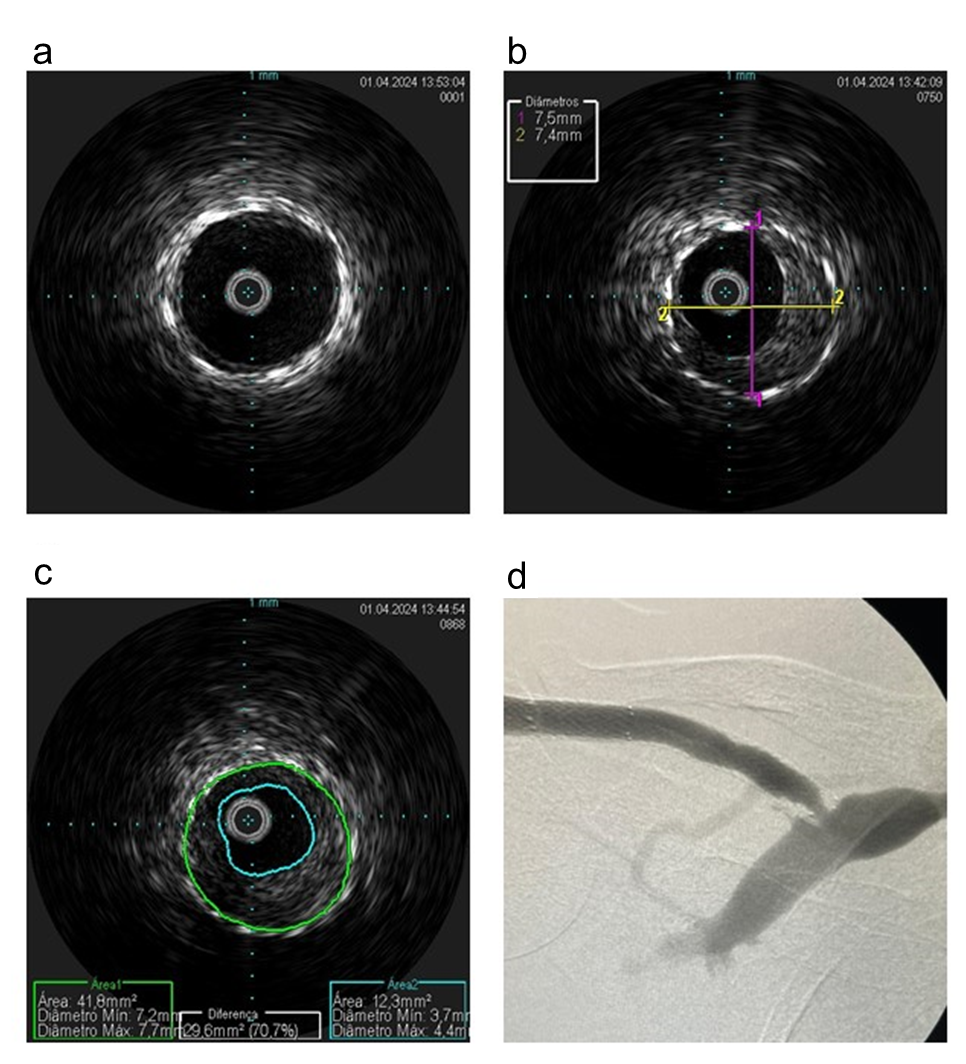


## Table S1. Target lesion primary patency according to sex.

| Sex | 1 month | 3 months | 6 months | 12 months |
| --- | --- | --- | --- | --- |
| Female | 100% | 93.23% | 78.43% | 62.50% |
| Male | 100% | 98.31% | 93.22% | 76.47% |
